# Awareness for People With Alzheimer’s Disease: Profiles and Weekly Trajectories

**DOI:** 10.3389/fnagi.2021.781426

**Published:** 2022-01-13

**Authors:** Amandine Mayelle, Capucine Hazebrouck, Mohamad El Haj, Daniel C. Mograbi, Pascal Antoine

**Affiliations:** ^1^Univ. Lille, CNRS, UMR 9193—SCALab—Sciences Cognitives et Sciences Affectives, Lille, France; ^2^Etablissements Pour Personnes Âgées Dépendantes La Colombe, Roncq and L’Orée du Monde, Halluin, France; ^3^Nantes Université, Univ Angers, Laboratoire de Psychologie des Pays de la Loire (LPPL—EA 4638), Nantes, France; ^4^Unité de Gériatrie, Centre Hospitalier de Tourcoing, Tourcoing, France; ^5^Institut Universitaire de France, Paris, France; ^6^Pontifical Catholic University, Rio de Janeiro, Brazil; ^7^Institute of Psychiatry, Psychology and Neuroscience, King’s College London, London, United Kingdom

**Keywords:** Alzheimer’s disease, anosognosia, awareness, temporal trajectory, profiles

## Abstract

**Objective:** To understand awareness and fluctuations of awareness in Alzheimer’s disease (AD), it is fruitful to consider the objects of awareness, e.g., cognitive functioning or recognition of the disease, as well as the mechanisms and modes of expression underlying awareness. With a holistic and discourse-centered approach, we aimed to identify different awareness profiles and test whether these profiles were stable or whether transitions from one profile to another occurred over short time intervals.

**Methods:** Twenty-eight residents of nursing homes with a diagnosis of AD participated in four semistructured interviews at biweekly intervals. These interviews were cluster analyzed to determine profiles of awareness. A Markov chain was applied to model their fluctuation.

**Results:** Five awareness profiles were observed that differed in terms of objects and underlying processes. Awareness proved to be quite stable for four of the five profiles. Interindividual variability in awareness was also observed through numerous different trajectories that were identified.

**Discussion:** Self-awareness and disease awareness are characterized by profiles that vary subtly between individuals. Fluctuations in awareness underscore the need to employ assessment intervals that closely reflect daily life in institutions.

## Introduction

Lack of awareness, also known as anosognosia, refers to the difficulties experienced by patients with certain neurological conditions, including Alzheimer’s disease (AD), in acknowledging their condition, symptoms and changes (Mograbi and Morris, 2018). According to Clare (2010), awareness in people with severe dementia is the ability to hold a reasonable or realistic perception or appraisal of, and/or respond accordingly to, a given aspect of their environment, situation, functioning or performance. Studies have sought to assess the progression of awareness of disease through cross-sectional or longitudinal designs.

The main finding of cross-sectional studies has been that lack of awareness increases as AD progresses (Starkstein et al., 2006; Maki et al., 2012; Mograbi et al., 2012; Conde-Sala et al., 2014; Baptista et al., 2019). A decline in awareness has been observed independent of the type of evaluation used (e.g., comparison of a patient and a relative, Starkstein et al., 2006; Maki et al., 2012; Conde-Sala et al., 2014; Baptista et al., 2019; the prediction-performance paradigm, Mograbi et al., 2012) as well as the stage of disease studied. However, the examination of a narrow time frame under a cross-sectional design does not allow potential fluctuations to be discerned over a stage of life that extends over years and months. To examine a time frame that more closely reflects what is experienced in institutions, some studies have employed a longitudinal perspective.

Similar to the cross-sectional studies, longitudinal studies have mainly observed an increased lack of awareness over time (McDaniel et al., 1995; Aalten et al., 2006; Clare and Wilson, 2006; van Vliet et al., 2013; Turró-Garriga et al., 2016; Sousa et al., 2017; Hanseeuw et al., 2020). This progressive loss of awareness is not the only pattern that has been observed. Indeed, Clare and Wilson (2006) and Turró-Garriga et al. (2016) observed a progressive loss of awareness for some participants and stability in awareness, albeit to a lesser extent, for others. Recently, Dourado et al. (2016) observed that nearly a quarter of the sample exhibited a deficit at the 1-year point but found an improvement in awareness for 12.3% of the sample. Taken together, these longitudinal studies indicate different patterns of change in awareness of disease. When the amount of time between measurements is reduced, the observed variation in patterns tends to increase, thereby raising the question of whether the intervals of assessment should be further reduced (e.g., monthly, weekly, or even daily follow-up). Some studies have reported the daily experiences of relatives (Clare and Woods, 2005; Walmsley and McCormack, 2017) and reported “moments of lucidness,” “flashes,” or alternation between “moments of presence and moments of absence” (Rozotte, 2001). In their qualitative study, Wawrziczny et al. (2016) highlighted the changing nature of the symptoms and the discourse of spouses with dementia about their symptoms. If the discourse of people with the disease evolves on a small time scale, a caregiving spouse can be expected to see changes from 1 day to the next, or family members visiting a loved one in a nursing home can be expected to see changes in discourse from week to week or month to month. These discourses may reflect varying awareness levels of the patient. To our knowledge, no study has measured awareness of disease at such short intervals.

Previous cross-sectional and longitudinal studies have had limitations, such as the use of different samples, in the comparison of stages and long intervals between evaluations. The occurrence of daily fluctuations reported in some studies was based only on comparative assessments. Indeed, another limitation is the quasi-systematic use of comparative methods to evaluate awareness, such as with the use of clinical ratings (McDaniel et al., 1995; van Vliet et al., 2013), the prediction-performance paradigm (Mograbi et al., 2012; Avondino and Antoine, 2015), and/or comparison of a patient’s assessment with that of a relative (Starkstein et al., 2006; Baptista et al., 2019; Hanseeuw et al., 2020). Although the information gained from such comparative methods is necessary and useful for understanding what the person with AD experiences, the perspective of the individual, what he or she understands about him- or herself and his or her evolution, and how this is expressed in his or her discourse deserve to be further explored. Finally, these evaluations tend to consider only one facet of awareness, namely, objects, the “what” that the evaluation is based on. In dementia research, several studies have even emphasized the need to distinguish between objects of awareness (Dourado et al., 2007; Markova et al., 2014), for example, by differentiating awareness of cognitive functioning and health condition, activities of daily living, emotional state, social functioning, and relationships (Lacerda et al., 2018). In the present study, objects refers to the basis of changes and new information (emotions, body, communication, autonomy, identity, cognitive abilities, memory, and AD) perceived by people with AD (Mayelle et al., 2019). Furthermore, O’Shaughnessy et al. (2021) stated that awareness is a multidimensional construct requiring a holistic approach. The phenomenon of awareness appears to be more than merely a “what” but rather a synergy associating the “what” (i.e., the objects of awareness) with the “how” in terms of the mechanisms and modes of expression underlying awareness (Mayelle et al., 2020).

Two key issues must be addressed. The first is the need to reduce the intervals of the observation of awareness to better approximate the patient’s experiences, including the investigation of objects, mechanisms, and modes of expression. The second is the need to obtain a sense of awareness as experienced by people with AD through a person-centered approach, i.e., based on their discourse reflecting their own perceptions and the meaning they assign to those perceptions. We propose two hypotheses. The first concerns the observation of different “profiles” of awareness characterized by the awareness of objects and the presence of mechanisms and modes of expression. While we hypothesize that both fully “aware” and “unaware” profiles will be observed, we also expect to observe more nuanced profiles that exhibit differences in terms of awareness of objects, mechanisms and modes of expression. The second hypothesis concerns fluctuations in awareness. Based on the patterns of fluctuations observed in longitudinal studies, we hypothesize that the profiles of awareness identified will exhibit three different types of patterns: deficit, stability, or improvement.

## Materials and Methods

### Design

This observational study was conducted at seven nursing homes in the Hauts-de-France region. Written consent was obtained for each participant. The ethics committee of the University of Lille approved the study (2018-267-S58).

### Participants

To be included in the study, the participants had to have resided at the nursing home for at least the previous 3 months. This inclusion criterion allowed the influence of adaptation to a new environment on awareness to be avoided. All participants had a diagnosis of AD dementia by an experienced neurologist or geriatrician based on the criteria of the National Institute on Aging–Alzheimer’s Association (McKhann et al., 2011). The participants also had to speak French or at least be capable of communicating in French for several minutes with the investigator.

The sample contained 28 participants [mean (M) age: 85.21; standard deviation (SD): 6.71]. Of the participants, 23 were women (aged 70–96 years, *M*: 86.04 years; *SD*: 5.83) and five were men (aged from 66 to 90 years, *M*: 85.25 years, *SD*: 5.25). The mean Mini Mental State Exam (MMSE) score was 13.68 (*SD*: 4.29). The majority of participants were at a moderate stage of dementia (*n* = 21). A minority were at a mild (*n* = 2) or severe (*n* = 5) stage.

### Procedure

Each participant was engaged in a series of four semistructured interviews based on systematic themes such as mood, emotions, well-being (physical and psychological), daily life, self-perception (body, personality), family, friends, relationship changes, cognitive functions, memory loss, elderly experience, disease and expectations for the future. The main questions were focused on personal experience, such as “How are you?” “What are you doing today?” and “Tell me about yourself.” Moreover, the investigator used mainly reformulations or repetitions. The objective of the interview was to follow only the participants’ experience and what they were able to say about it. For each participant, all four interviews were conducted, transcribed and rated by one of two trained psychologists.

### Measure

#### Mini Mental State Exam

Cognitive functioning was assessed with the MMSE (Folstein et al., 1975), which is a test of spatiotemporal orientation, attention and calculation as well as memory, language and visual construction.

#### Awareness of Self and Disease Assessment

The Awareness of Self and Disease Assessment (ASDA) scale provides an evaluation of awareness of self and the disease that is centered on the person with AD (for a detailed description, see Mayelle et al., 2019). The ASDA is designed to be as close as possible to the subjective experience of having the disease. Each interview carried out with the ASDA was evaluated based on 22 items (see Table 1) in three categories: objects (9), mechanisms (5), and modes of expression (8). Objects refers to the basis of changes and new information (emotions, body, communication, autonomy, identity, cognitive abilities, memory, and AD) perceived by people with AD. Mechanisms refers to processes of awareness (e.g., observation of the environment, perception of the expressions of others, comparison between the past and the present, metacognition and confrontation with difficulties). Modes of expression are how people express their awareness (denial, expression of doubts, expression of changes with a causal attribution, a self-description, a self-assessment or a need).

**TABLE 1:** The 22 items of the Awareness of Self and Disease Assessment (ASDA).

**Objects**	1. Environment 2. Emotions 3. Body 4. Communication 5. Autonomy 6. Identity changes 7. Loss of cognitive abilities 8. Memory 9. Disease	Changes in the environment All new emotions Changes in sensations and physical abilities Difficulties with verbal treatment information and verbalization Difficulties during activities of daily living Personality/mental/social status changes Difficulties in concentration and location in space and time Difficulties in learning and remembering information Awareness of being a person with Alzheimer’s disease

**Mechanisms**	1. Observation of the environment 2. Perception of the looks of others 3. Comparison between the past and the present 4. Metacognition 5. Confrontation of difficulties	Awareness of changes with environment observation Awareness of changes in the looks/discourses/actions of others Awareness of differences in physical and psychological state and loss of independence and autonomy Discourse on changes during a metarepresentation/self-analysis Awareness of changes by observation of decreased physical and psychological abilities

**Modes of expression**	1. Denial 2. Bewilderment 3. Attribution 4. Description 5. Judgment 6. Recognition of the need for help 7. Use of coping strategies 8. Confirmation of the disease	Opposition, denial of changes and/or causes Expression of doubts/hesitations about daily life and the future Expression of changes with a causal attribution Expression of changes with a self-description Expression of changes with a self-assessment Expression of changes in recognizing the need for help during activities of daily living. Expression of changes by using coping strategies Expression of changes by recognizing Alzheimer’s disease

Each item of the mechanisms and modes of expression categories was rated on a six-point Likert scale (1: “Minimally present,” 2: “Slightly present,” 3: “Mildly present,” 4: “Moderately present,” 5: “Strongly present,” 6: “Extremely present”). Each item of the object category was also rated according to a six-point Likert scale (1: “Strong unawareness,” 2: “Mild unawareness,” 3: “Slight unawareness,” 4: “Slight awareness, 5: “Mild awareness,” 6: “Strong awareness”). A high rating was associated with a high level of awareness. For each category, the overall score, between 1 and 6, is the mean of its items. Cronbach’s alpha was high (from0.77 to 0.86). The ASDA does not presently provide a cutoff score; rather, the objective is to create a “profile of awareness” for each person with AD. The ASDA is a subjective measure based only on what the participant is able to say. Consequently, this method resulted in missing values, recorded as “Not evaluated.” Overall, the rate of missing values was 14.85%, reflecting objects, mechanisms or modes of expression that could not be scored during an interview.

### Data Analysis

The statistical analyses were carried out with R software (version 3.5.2). As a preliminary step, the “FactoMireR” and “MissMDA” packages allowed the description of the data and imputation for the missing values. A cluster analysis [i.e., a hierarchical ascendant classification (HAC)] was carried out to determine the different profiles of awareness. The aim was to ensure that the interviews within a profile were as similar as possible and that the profiles were as contrasting as possible. To do so, the HAC was used to measure the similarity (or, conversely, the distance) between the interviews in pairs based on the scores of the 22 items. All these measures together constitute a distance matrix. Two identical interviews will have a distance of zero. The more different the interviews are, the greater their distance. The HAC thus makes it possible to iteratively position the interviews in relation to each other to produce a dendrogram. The classification is hierarchical because it produces increasingly larger profiles, including subgroups within them. This dendrogram is then analyzed to produce the most easily interpretable organization of profiles.

The second analysis used a Markov chain method and was performed with the “Markovchain” package. This analysis allowed the probabilities of a transition from one profile to another to be quantified and modeled. Awareness is considered to be a dynamic system composed of states and transitions between these states. The states were defined by the profiles identified in the previous analysis, and we then attempted to measure the probability that each state (profile) would remain stable the next time or evolve toward any of the other possible states (profiles).

## Results

### Profiles of Awareness

The analyses included all 112 interviews conducted with the 28 residents. We identified five clusters (Table 2).

**TABLE 2:** Summary of the clusters derived from the hierarchical ascendant classification.

		HAC				
		Profile 1	Profile 2	Profile 3	Profile 4	Profile 5
Number of interviews		18	27	28	21	18
Mechanisms	*Mean*	4.44	2.37	3.35	4.73	1.94
	*SD*	0.511	0.49	0.58	0.711	1.17
Objects	*Mean*	3.73	3.15	2.77	4.56	1.98
	*SD*	0.47	0.47	0.37	0.45	0.58
Modes of expression	*Mean*	3.01	2.43	1.98	3.92	1.59
	*SD*	0.60	0.52	0.36	0.65	0.63

Profile 1 was characterized by a high presence of mechanisms (*M* = 4.44, *SD* = 0.511), a moderate presence of modes of expression (*M* = 3.014, *SD* = 0.603) and a moderate awareness of objects (*M* = 3.728, *SD* = 0.469). Profile 2 was characterized by a low presence of mechanisms (*M* = 2.37, *SD* = 0.489), a moderate presence of modes of expression (*M* = 2.426, *SD* = 0.516), and a moderate awareness of objects (*M* = 3.148, *SD* = 0.465). Profile 3 was characterized by a moderate presence of mechanisms (*M* = 3.35, *SD* = 5.83), a low presence of modes of expression (*M* = 1.982, *SD* = 3.56), and a low awareness of objects (*M* = 2.77, *SD* = 0.367). Profile 4 was characterized by a high presence of mechanisms (*M* = 4.733, *SD* = 7.11) and modes of expression (*M* = 3.923, *SD* = 0.65) as well as a high level of awareness of objects. This profile represented a preserved awareness of self and disease. Profile 5 was characterized by a low presence of mechanisms (*M* = 1.944, *SD* = 1.17) and modes of expression (*M* = 1.59, *SD* = 0.625) and a low level of awareness of objects. Profile 5 represented a lack of awareness and thus was the opposite of profile 4.

### Fluctuations in Profiles as Modeled by a Markov Chain

**FIGURE 1:**
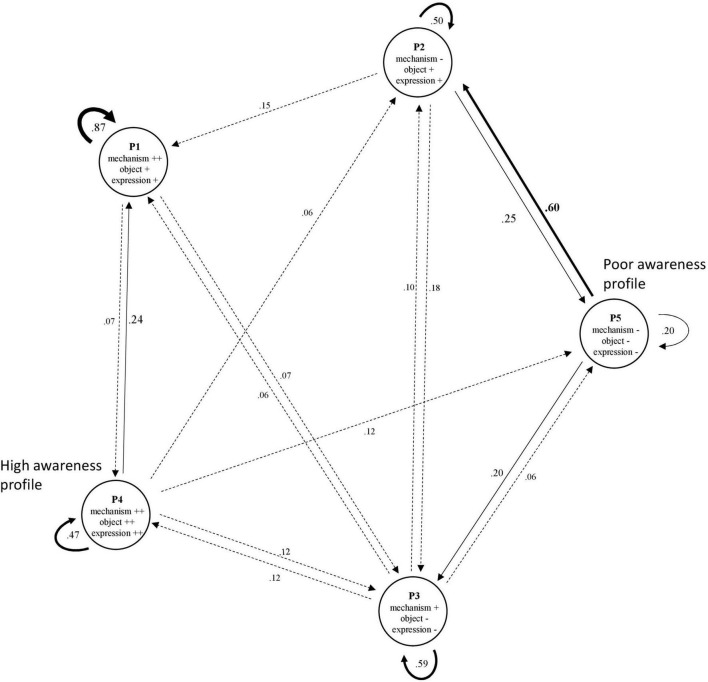
Modeling of fluctuations in profiles of awareness by Markov chain. P, Profile; Mech, Mechanisms; Exp, Modes of expression. For ease of reading, the connections are coded according to the probability of the transition (dashed < 0.2; normal < 0.4 and bold > 0.4), as are the font sizes of the coefficients. The absence of a connection between two profiles is explained by the low occurrence or absence of a transition.

The Markov chain used to model the biweekly fluctuations in the awareness of self and the disease is represented in Figure 1.

The modeling showed that over the 2-week period, awareness proved to be quite stable for four of the five profiles [probability (P) from 0.47 to 0.87], particularly for individuals who made abundant use of mechanisms, who made moderate use of modes of expression and who had a moderate level of awareness. In contrast, the profile corresponding to the lowest level of awareness appeared to be more transient and to have a low probability of stability (*P* = 0.20).

Furthermore, numerous intercluster transitions were observed. For example, profile 3 (moderate use of mechanisms, low use of modes of expression, and a low level of awareness of self and disease) appeared as a stable profile while connected with each of the other profiles (*P* = 0.06–0.20). The profiles tended to be connected with one another; that is, individuals passed from one profile to another, either in a bidirectional manner, such as between profile 4 and profile 3 (*P* = 0.12), or in a more unidirectional manner, such as between profile 4 and profile 1 (*P* = 0.07 and 0.24). Regarding the latter example, a person had a greater probability of changing from profile 4 to 1 than from profile 1–4. The highest probability of changing from one profile to another was between profile 5 and profile 2 (*P* = 0.60). Beyond the stability of their profiles, the participants in the study had a high probability of progressing from a low level of awareness of disease (with low use of both mechanisms and modes of expression) toward a low/moderate level of awareness (with moderate use of modes of expression and low use of mechanisms).

Finally, some profiles, such as profile 5 and profile 1, did not share a relationship,. That is, a person with a low level of awareness of disease (with low use of mechanisms and modes of expression) did not progress to a moderate level of awareness (with moderate use of modes of expression and a high level of use of mechanisms), and vice versa.

### Focus on Individual Weekly Fluctuations in Awareness

It was possible to observe individual fluctuations for each of the 28 participants. Table 3 shows the profile assessed at the four interview times for each resident and, in summary, their number of profiles and number of transitions.

**TABLE 3:** Individual fluctuations for each of the participants in the four stages.

Participants	Time 1	Time 2	Time 3	Time 4	Nb of profiles	Nb of changes
1	2	2	2	2	1	0
2	2	2	2	2	1	0
3	3	3	3	3	1	0
4	3	3	3	3	1	0
5	4	4	4	4	1	0
6	5	5	5	5	1	0
7	1	1	4	4	2	1
8	3	4	4	4	2	1
9	4	4	5	5	2	1
10	4	4	4	2	2	1
11	5	5	2	2	2	1
12	5	3	3	3	2	1
13	1	1	3	1	2	2
14	2	2	5	2	2	2
15	2	5	2	2	2	2
16	3	1	1	3	2	2
17	3	3	2	3	2	2
18	5	3	5	5	2	2
19	4	1	4	1	2	3
20	5	2	5	2	2	3
21	1	1	2	3	3	2
22	1	1	4	3	3	2
23	2	2	3	1	3	2
24	3	1	1	4	3	2
25	3	2	5	3	3	3
26	4	2	1	4	3	3
27	4	3	2	3	3	3
28	3	5	2	1	4	3

The main finding is the interindividual variability in the evolution of awareness, with 24 distinct trajectories observed.

Complete stability (i.e., 0 transitions) was observed in six participants (approximately 21% of the sample) in profiles 2–5. In contrast, 6 participants switched at each interview among 2–4 profiles. The remaining 16 residents (57%) made one (*n* = 6) or two (*n* = 10) transitions.

## Discussion

Research on awareness in AD provides information regarding its heterogeneity and temporality. However, it does not allow appreciation of the heterogeneity of the processes of awareness (Mayelle et al., 2019, 2020) and daily fluctuations in awareness. The first part of this work involved the observation of different profiles of awareness characterized by the awareness of objects and the presence of mechanisms and modes of expression. The second part of this work involved the observation of weekly fluctuations in these aspects.

We first hypothesized that profiles of awareness would range from extremes (i.e., aware vs. unaware) to more mixed profiles. We observed five different profiles, two of which indicated complete awareness and lack of awareness. The other three profiles differed mainly in terms of the use (i.e., frequency and adaptation) of mechanisms and modes of expression of awareness. Based on these results, we were able to confirm that awareness in AD is heterogeneous, highlighting distinct levels of awareness both in terms of objects (the “what”) and processes (the “how”) that characterize the disease (Mayelle et al., 2020).

In the second part of the study, our results were consistent with prior results, and they provide evidence of the non-linearity of awareness (Lacerda et al., 2020). While we were able to show that each participant exhibited a unique temporal trajectory of fluctuation(s) in awareness, the analysis of the transitions between the profiles revealed a number of trends. We observed that the profiles of awareness tended to remain stable between measurement times. However, the profiles could improve or worsen for certain components (i.e., objects, mechanisms, and/or modes of expression). Furthermore, while a high general level of awareness could become very low in the short period between measurements, a low level of awareness could not become very high.

Similarly, we noted that the highest probability of change was related to a slight improvement in the level of awareness of objects and the presence of modes of expression. For this particular fluctuation, we proposed three interpretations.

First, one perspective on this improvement is the concept of the petrified self (Mograbi et al., 2009). It is possible that when confronted with a mistake or criticism, a resident became temporarily aware of his or her condition or its evolution. However, the long-term integration of this new information would fail, resulting in a return to a lower level of awareness at the next interview.

Second, after returning to the interviews, we assumed that this fluctuation was specifically linked to events and/or changes in the environment and the participant’s daily life (e.g., a room change). A person exhibits a specific profile of awareness that is influenced by an environmental change. Once this change becomes established, the person returns to the initial level, which translates into a deficit followed by an improvement. This interpretation particularly reflects changes in the integration and rejection of the disease as characteristic of the self (Pearce et al., 2002; Frazer et al., 2012). O’Shaughnessy et al. (2021) suggested that people with severe AD may not demonstrate awareness, not because they are unable to but rather because environmental factors are not conducive to expressing awareness.

Third, the relevant interviews tended to highlight the influence of the investigator and his or her attitude toward awareness. When a person has disorganized speech, the practices of reformulation, the verbalization of a thought, or the stimulation of verbal exchanges appear to help him or her express him- or herself in a more suitable and coherent manner, thereby restoring meaning (Ducato et al., 2013). According to the ASDA (e.g., Table 1), this process can be translated, for example, into the expression of changes with a self-description, the expression of doubts about daily life and the future, or the expression of a need for help. To best verify this effect, the ASDA could be made part of an interventional protocol employing dignity therapy (Martínez et al., 2017) or validation therapy (Neal and Barton Wright, 2003). These two approaches aim to support the person with AD with caring verbal expression to encourage him or her to integrate these events and give them meaning. A pre- and postintervention ASDA evaluation of awareness could quantify the effect of others’ attitudes on awareness.

Our data allowed the modeling of fluctuations in awareness of disease that have often been studied from the perspective of patients’ relatives (Rozotte, 2001; Clare and Woods, 2005; Walmsley and McCormack, 2017). The presentation of these profiles and their possible fluctuations to health professionals could allow these professionals to verbalize their daily experience in care and help them understand the relative instability of the patient’s perspective and therefore the need to repeat the interactions to understand the extent of that fluctuation. For example, the implementation of the methodology employed in the present study on a case-by-case basis in residential care facilities could allow caregivers to understand and then adjust to the reactions of people with AD while they provide support. Indeed, caregivers evolve in a reality that differs from that of the person with AD (Hertogh et al., 2004), and they can find themselves powerless when confronted with a refusal of care or only partial observance of treatment for which they do not understand all of the underlying processes (Fischer et al., 2019).

However, increasing the sample size appears to be necessary to reinforce the validation of the identified profiles of awareness. Such an increase in the sample size would also allow more clusters and hence more nuances in the profiles to be identified. Furthermore, an increase in the sample size would allow confirmation and increase the generalizability of the possible trajectories observed. Although the sample reflects the overrepresentation of women living in nursing homes, an increase in the number of male participants would allow verification of the impact of gender on awareness (Liu et al., 2017) and the distribution of the profiles. Indeed, the limitations of the study are linked mainly with the characterization of the sample and the impact of these parameters on the profiles of awareness and their possible fluctuations. As this was a pilot study, we essentially collected data centered on awareness without collecting information regarding education level, time since diagnosis or time living in a nursing home, the severity of the disease, neuropsychiatric symptoms (Yoon et al., 2017) or personality (Rankin et al., 2005). Now that this study has shown fluctuations in awareness, it is important to control, for example, the cognitive profile of the participants to understand the role played by these variables in the awareness profiles.

## Conclusion

This study aimed to understand the awareness of self and disease in people with AD by adopting a perspective based on profiles rather than a single score. These profiles were studied on the basis of their fluctuations from a restricted temporal perspective. Multiple profiles and trajectories were identified, illustrating inter- and intraindividual variability in awareness. These results confirm the need to focus on the subjective experience of the person with assessment intervals that closely reflect his or her daily life.

## Data Availability Statement

The raw data supporting the conclusions of this article will be made available by the authors, without undue reservation.

## Ethics Statement

The studies involving human participants were reviewed and approved by the Ethics Committee of the University of Lille (2018-267-S58). The patients/participants provided their written informed consent to participate in this study.

## Author Contributions

AM and PA were responsible for the conception and design of the study and responsible for the drafting of the manuscript. AM, CH, MEH, DM, and PA contributed to the collection and analysis of data. All authors critically revised the draft and approved the final version.

## Conflict of Interest

The authors declare that the research was conducted in the absence of any commercial or financial relationships that could be construed as a potential conflict of interest.

## Publisher’s Note

All claims expressed in this article are solely those of the authors and do not necessarily represent those of their affiliated organizations, or those of the publisher, the editors and the reviewers. Any product that may be evaluated in this article, or claim that may be made by its manufacturer, is not guaranteed or endorsed by the publisher.
